# Isolation and passaging of reptarenaviruses utilizing cultured snake cells suggest tissue tropism and restrictions in segment reassortment

**DOI:** 10.1099/jgv.0.002154

**Published:** 2025-10-08

**Authors:** Annika Lintala, Udo Hetzel, Leonora Szirovicza, Emilia Timin, Anja Kipar, Jussi Hepojoki

**Affiliations:** 1 Faculty of Medicine, Medicum, Department of Virology, University of Helsinki, Helsinki, Finland; 2 Vetsuisse Faculty, Institute of Veterinary Pathology, University of Zürich, Zürich, Switzerland; 3 Faculty of Veterinary Medicine, Department of Veterinary Biosciences, University of Helsinki, Helsinki, Finland

**Keywords:** infection, reptarenavirus, segment rearrangement, tropism

## Abstract

Reptarenaviruses cause boid inclusion body disease that can affect the fitness of the infected animals through a variety of clinical signs. Reptarenaviruses infect most tissue types in the affected individuals and spread efficiently in captive snake collections. Their genome consists of a small (S) and a large (L) segment, and the reptarenavirus-infected snakes often carry multiple genetically divergent reptarenavirus S and L segments, suggesting reptarenavirus coinfections occur frequently. We previously observed that reptarenavirus S and L segment combinations may vary between the tissues of an infected snake, leading to the hypothesis that the segment combination might contribute to tissue and/or species tropism. To test the hypothesis, we inoculated various cell lines derived from different tissues of several constrictor snake species with two samples containing multiple reptarenavirus segments (F15, two S and seven L segments; F17, one S and four L segments). We blind-passaged both virus samples five times in each cell line and monitored the presence of the segments in the supernatants through reverse transcription PCR. We also passaged the cells following the first inoculation with F17 and studied the segments present as above. The analysis revealed that some L segments were only present in supernatants with a specific S segment, suggesting preferred S and L segment pairs, thereby arguing against free reassortment of the segments. The results also showed that boa constrictor-derived cell lines supported reptarenavirus infection slightly better than pythonid-derived cell lines.

## Introduction

In virology, tropism refers to either the species or range of organs, tissues or cells in which a given virus can successfully undergo its replication cycle [1,2]. Receptor availability, intracellular environment and mechanisms to evade the immune system are some of the factors that affect the ability of a virus to infect a cell in a tissue of a given organ in a certain susceptible host species [1,2].

Members of the genus *Reptarenavirus* within the *Arenaviridae* family [3,4] cause boid inclusion body disease (BIBD) in boid and pythonid snakes [5]. Snakes with BIBD can show variable clinical signs, including anorexia and various central nervous system signs [6,8]. They are also claimed to be more susceptible to secondary infections due to immunosuppression [1,2]. This could be related to infection of the immune cells [9].

The reptarenavirus genome is bisegmented, the small (S) segment encodes for glycoprotein precursor and nucleoprotein (NP) and the large (L) segment encodes RNA-directed RNA polymerase (RdRp) and matrix/Z protein (ZP) [4]. Cytoplasmic inclusion bodies (IBs), predominantly composed of reptarenavirus NP, are found in almost all tissues and cell types of the affected animals [5,6, 10, 11]. BIBD diagnosis is based on the detection of IBs in blood smears or tissue biopsies [6]. A large proportion of snakes with reptarenavirus infection and detectable IBs, hence being diagnosed with BIBD, are clinically healthy but expected to become clinically ill at a later stage [12,13]. Experimental infections support the fact that reptarenaviruses cause persistent infection in the snakes [7,8]. Snakes with BIBD often carry a swarm of genetically divergent reptarenavirus S and L segments at unbalanced ratios (more L segments) [12,14, 15] and fairly frequently also hartmaniviruses (another arenavirus genus), the potential role of which in BIBD has not been clarified [16,17]. We studied the segment accumulation previously by establishing persistently reptarenavirus-infected cell cultures [18] that we further utilized for conducting superinfection experiments [19]. These provided evidence that superinfections of persistently infected captive snakes would have contributed to the frequent coinfections observed in BIBD-positive animals [19].

Descriptions of BIBD among captive snakes date back to the 1970s [10]. Reports on BIBD in captive boas in their native regions are rare; however, we recently described several cases both in Costa Rica and Brazil [20,21], including free-ranging Costa Rican boas [20]. Mammarenaviruses have specific natural hosts in which the virus causes a persistent infection, e.g. *Mus musculus* for lymphocytic choriomeningitis virus (LCMV) and *Mastomys* sp*.* for Lassa virus [22,23]. The species specificity and evolutionary codivergence of mammarenaviruses and their hosts indicate coevolution [24]. Interestingly, affected boa constrictors in both Brazil and Costa Rica harboured different sets of reptarenaviruses [20,21], which appear to argue against reptarenavirus coevolution with *Boa constrictor*, thereby suggesting that boas might not be the natural host of reptarenaviruses. Furthermore, the wide variety of reptarenavirus S and L segments observed in boa constrictors [11,12, 14, 15] argues against strict species specificity for reptarenaviruses. Detection of reptarenaviruses and BIBD in other boid species [6,20, 25,27] and pythons [6,13, 26, 28, 29] also speaks against a host species specificity similar to that of mammarenaviruses. Studies dating back as far as the 1930s have shown that a single LCMV isolate could infect multiple tissues in white mice [30], similar to what is observed for reptarenaviruses in BIBD-positive animals [7,8, 11, 12, 14, 16, 19, 31]. However, in an earlier study, we found a snake with several reptarenavirus S and L segments in the blood, while only a single S and L segment pair was detected in the brain tissue [12]. This observation led us to speculate that reptarenavirus S and L segment combinations could be drivers of tissue and/or species tropism. To study the hypothesis, we used two reptarenavirus tissue samples with several S and L segments to inoculate a panel of cell cultures derived from different organs of boas and pythons through spontaneous immortalization. We hypothesized that passaging of the virus would lead to the propagation of the reptarenavirus S and L segment pairs that are most viable in each cell line, thereby revealing potential species or tissue tropism. In addition, with the other tissue homogenate, we compared virus isolation and propagation by passaging the inoculated cells with the idea that such an approach might help in propagating low abundance or slowly replicating segment combinations, and perhaps even lead to a persistent infection, as observed with other reptarenaviruses [18,19].

## Methods

### Virus segments, cell lines and passaging experiments

Tissue samples with more than a single reptarenavirus S and L segment pair (Table 1) served to inoculate snake cell lines, one derived from blood (F15) [12] and the other from brain (F17) [16] of two different boa constrictors with BIBD. F15 included L segments of seven reptarenaviruses, keijut pohjoismaissa virus 1 (KePV-1), kuka mitä häh virus 1 (KMHV-1), grüetzi mitenant virus 1 (GMV-1), aurora borealis virus 4 (ABV-4), tavallinen suomalainen mies virus 2 (TSMV-2), suri vanera virus 2 (SVaV-2) and University of Helsinki virus 4 (UHV-4), and S5-like and S6-like reptarenavirus S segments (Table 1). F17 included L segments of four reptarenaviruses, hipoen jatkoon virus 1 (HJV-1), peto jauhoksi virus 1 (PJV-1), KMHV-1 and mistä näitä tulee virus 1 (MNTV-1), and S7-like reptarenavirus S segment (S7-like). The F17 swarm also included a Hartman virus [veterinary pathology Zurich virus 1 (VPZV-1)] (Table 1).

**Table 1.** Virus segments The information for genus, abbreviation and GenBank accession references is provided for each virus segment present and used in the studies. Each forward and reverse primer was designed based on the sequence. Abundances of each segment in the F15 and F17 samples used for initial inoculation are presented in percentages.

F15 virus segment	Genus – segment	Abbreviation	GenBank accession no.	Forward primer	Reverse primer	% of segments in NGS
University of Helsinki virus 4	Reptarenavirus – L	UHV-4	KX527587	CATTCTTTCAGGATCAAAATAATC	GAAAGTAAAATTGAGCCTCCAG	1.4
Tavallinen suomalainen mies virus 2	Reptarenavirus – L	TSMV-2	KX527591	CTTTGAGGGTCATAATAATC	CTGAATCAGAAATTGGGAAGC	18.8
Aurora borealis virus 4	Reptarenavirus – L	ABV-4	KX527592	CAATCTGCTTGGTTCATAATAATC	CTGTGGAGTTGAGGGTGAAT	2.2
Suri Vanera virus 2	Reptarenavirus – L	SVaV-2	KX527587	CTTTTAGGGTTGTAGTAATCAACTAAA	CAGTCTGCGCTGTTGGA	26.4
Keijut pohjoismaissa virus 1	Reptarenavirus – L	KePV-1	KX527589	GTCGTTGAGACCTAGAAGG	CTGTACTTACAAAACCAGTCAA	7.5
Grüetzi mitenant virus 1	Reptarenavirus – L	GMV-1	KX527593	GCACGATGGGCTTCAAGT	AAGGGTGATGGAACATTTCTG	0.8
Kuka mitä häh virus 1	*Reptarenavirus* – L	KMHV-1	KX527588	TCGTCTGATCCCAGATGT	GCTTTTGATGAGACACTCCT	1.3
S6-like virus	Reptarenavirus – S	S6-like	KX527578	ATAAGGTCAGGGTATAACTTGG	GAACTTGGCATAAAAATACAAATAAATG	36.7
S5-like virus	Reptarenavirus – S	S5-like	KX527579	GTCAGGATAGAGTCTGGGAGCAT	TGAACATTCAGAGGGAATTTGGCATC	4.9
**F17 virus segments**	**Genus – segment**	**Abbreviation**	**GenBank accession No.**	**Forward primer**	**Reverse primer**	**% of segments in NGS**
Hipoen jatkoon virus 1	Reptarenavirus – L	HJV-1	MH483085	AGGGCACACAATCAAACTTAC	TTCAAGGCACATTCCATACAG	30.2
Peto jauhoksi virus 1	Reptarenavirus – L	PJV-1	MH483086	CTCCCATCAAATACAGACAGAC	GGGCTAGAATCTAAAGCTGAAC	26.8
Kuka mitä häh virus 1	Reptarenavirus – L	KMHV-1	MH483084	TCTTCTACACCAACACCCC	GTTCTGATTGAAAATCCACCAC	17.3
Mistä näitä tulee virus 1	Reptarenavirus – L	MNTV-1	MH483087	TCACATCACCTTGAATGACAG	GATTTCTGCAAATGGTGATCTAG	14
Veterinary Pathology Zurich virus 1	Hartmanivirus – L	VPZV-1	MH483040	GCTTAGTCTAGCCAAGAGTCC	CCACAAAAGCAGGCAATATTC	0.6
S7-like virus	Reptarenavirus – S	S7-like	MH483088	GCAATATCATTTAGGGCTTCC	GATCCAGACCTAAGCTAAGTG	9.3
Veterinary Pathology Zurich virus 1	Hartmanivirus – S	VPZV-1	MH483039	GGTATTCGTCTAAATGGGAGC	CCTGGCATTTTCTGGTCATTAC	1.9

We utilized previously established cell lines originating from *B. constrictor* kidney (I/1Ki and V/1Ki), brain (V/4Br), lung (V/4Lu and V/5Lu), liver (V/1Liv) and heart (V/2 Hz) and *Morelia viridis* brain (VII/2Br) and liver (VII/2Liv) (Table 2). In addition, U.H. established two new cell lines, as described in [5], from *Morelia spilota* liver (IX/1Liv), and *Python regius* heart (VI/1 Hz) (Table 2). The cell lines were confirmed to be reptarenavirus-free by immunofluorescence and immunohistochemical staining using a broadly cross-reactive antiserum against reptarenavirus nucleoprotein, as described in [21]. The morphology of the cell lines was recorded with a Nikon Eclipse Ti (Nikon, Basel, Switzerland) inverted microscope (Fig. S1, available in the online Supplementary Material). The Roman numeral in the name indicates the clutch of snakes and the Arabic number is an identifier for an individual snake from the specific clutch. We maintained the cells at 30 °C with 5% CO_2_ in Minimal Essential Medium supplemented with 10% FBS, 200 mM l-glutamine, 100 µg ml^−1^ of streptomycin and 100 U ml^−1^ of penicillin.

**Table 2.** Cell line origins Detailed information on the cell line origin: snake species and tissue type. Tissue samples used for inoculation are showcased with each cell line. Cells from previous studies are presented first, and cell lines established in this study are described in the last two rows.

Cell line origin: family – species	Tissue origin	Abbreviation	Inoculated with	Reference
*Boidae – B. constrictor*	Kidney	V/1Ki	F15	[33]
*Boidae – B. constrictor*	Lung	V/5Lu	F15	[33]
*Boidae – B. constrictor*	Kidney	I/1Ki	F15, F17	[5]
*Boidae – B. constrictor*	Liver	V/1Liv	F15, F17	[33]
*Boidae – B. constrictor*	Heart	V/2 Hz	F15, F17	[33]
*Boidae – B. constrictor*	Brain	V/4Br	F15, F17	[32]
*Boidae – B. constrictor*	Lung	V/4Lu	F17	[32]
*Pythonidae – M. viridis*	Brain	VII/2Br	F15, F17	[32]
*Pythonidae – M. viridis*	Liver	VII/2Liv	F15, F17	[32]
*Pythonidae – P. regius*	Heart	VI/1 Hz	F15, F17	Established in this study
*Pythonidae – M. spilota*	Liver	IX/1Liv	F15, F17	Established in this study

**Fig. 1.**
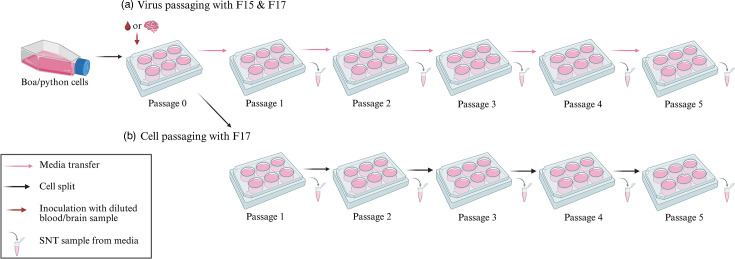
Workflow of passaging viruses and cells. (**a**) The cells were split (black arrow) on 6-well plates the day before the inoculation with tissue samples containing virus segments (red arrow). Media samples (microtube) were collected before each virus passage (pink arrow), where the media was inoculated with newly plated cells. (**b**) Cells inoculated with the F17 sample were split and passaged five times. Media samples were collected before each passage.

For the inoculation, we diluted the samples F15 (blood) and F17 (brain homogenate) 1 : 10 in the fully supplemented culture medium and allowed the viruses to adsorb onto cells for 1 h (Fig. 1a). After the incubation, we washed the cells with fully supplemented culture medium, placed them back into the incubator supplemented with fresh medium and collected the supernatant (SNT) samples (Fig. 1a) 10 to 14 days post-inoculation. The SNT served as the inoculum for the next passage of cells (Fig. 1a), referred to as viral passaging, which we repeated up to five times. In parallel, we performed five rounds of passaging of the cells initially inoculated with the F17 brain sample (Fig. 1b). When passaging the cells, we first removed the growth medium, washed the cells with 2 ml of 0.25% Trypsin-EDTA solution (Sigma-Aldrich) to remove residual medium, added 1.5 ml of fresh trypsin and incubated at 30 °C until they all had detached. We then made a cell suspension with fully supplemented fresh growth medium and moved a portion of the cells into clean flasks and incubated the cells at 30 °C with 5% CO_2_ until the next round of passaging.We collected samples of the SNT prior to each passage and stored the collected SNTs at −80 °C until analysed by reverse transcription PCR.

### Reverse transcription PCR

We used the sequences available from the earlier studies [12,16] to design primers (from Metabion) for each of the segments (Table 1). GeneJet RNA Purification Kit (Thermo Fisher Scientific) served for RNA isolation and RevertAid First Strand cDNA Synthesis Kit (Thermo Fisher Scientific) with Random Hexamer primers (Thermo Fisher Scientific) for transcription of cDNA, all according to the manufacturer’s instructions. Phusion Flash High-Fidelity PCR Master Mix (Thermo Fisher Scientific) was used at 10 µl volume [5 µl Phusion Flash Master Mix, 0.625 µl of both forward and reverse primers (final concentration 0.5 µM), 1 µl of the cDNA and 2.75 µl of H_2_O] and served for PCR amplification of the segment pieces on a Techne 3prime thermal cycler (1, denaturation: 10 s at 98 °C; 2, denaturation: 1 s at 98 °C; 3, annealing: 5 s at 54, 59 or 63 °C, depending on the primer pair; 4, extension: 7 s at 72 °C; steps 2 to 4 were repeated 30 times, and final extension 1 min 72 °C; cool down at 4 °C). We then detected the presence or absence of L and S segment PCR products through standard agarose gel electrophoresis.

## Results

In a previous study on the vertical transmission of reptarenaviruses, we identified a boa constrictor with BIBD that carried a pair of reptarenavirus S and L segments in the brain, while a swarm of two S and seven L segments was present in the blood [12]. This finding led us to hypothesize that reptarenavirus segment combinations could mediate tissue tropism. To study the hypothesis, we selected a panel of *B. constrictor* cell cultures originating from different tissues (kidney, lungs, heart, brain and liver) utilized in our earlier studies [5,32, 33]. While mainly observed in *B. constrictor*, BIBD also affects boid snakes of the family *Pythonidae*, often with a more severe clinical course, including faster and more severe signs in the animals [6,10]. Therefore, to study potential species tropism, we also utilized two cell lines established from *M. viridis* liver and brain in our earlier study [32] and generated one cell line each from the heart of a *P. regius* and from the liver of an *M. spilota*. The cell cultures were established in neutral media, i.e. without any compounds that could drive specific differentiation (these are not available for reptile cells) and yielded rather undifferentiated appearing roundish or elongate to spindle-shaped adherent cells (Fig. S1), with the exception of the boa constrictor brain cell line (V/4Br) that exhibited large cells reminiscent of neurons, and the boa constrictor heart cell line (V/2 Hz) which contained individual cells reminiscent of myocytes (Fig. S1A). Further characterization of the cells has not been possible so far due to a lack of cross-reacting antibodies against cell-specific markers, and reverse transcription PCR-based characterization approaches are limited due to a lack of fully annotated genomes for the snake species included. The python-derived cell lines were comprised of cells that generally appeared more roundish and were slightly smaller than the boa constrictor-derived cell lines (Fig. S1B–C).

We inoculated the cell lines with either blood, F15 (two S segments and seven L segments) [12], or a brain homogenate, F17 (one S and four L segments, plus a pair of hartmanivirus segments) [16] from boa constrictors with BIBD (Table 1). To study whether specific reptarenavirus S and L segment combinations would be enriched in cell lines originating from certain tissues or snake species, thereby implying tropism, we followed the transmission of segments up to five passages. In addition, we studied the effect of passaging of the inoculated cells with F17 brain homogenate for five cell passages (Fig. 1b). In all cell lines, the cell morphology remained stable throughout the passaging and inoculations. We did not observe any alterations in cell morphology following inoculations in any of the cell lines that would suggest a cytopathic effect.

The results with F15 inoculations showed all boa constrictor cell lines to support propagation of most of the reptarenavirus segments present in the blood sample (Table 3). The brain- (V/4Br), heart- (V/2 Hz) and lung-derived (V/5Lu) cell lines each allowed propagation of two S segments (S5-like and S6-like S segments) and four L segments (TSMV-2, SVaV-2, ABV-4 and KePV-1 L segments). However, the kidney-derived (I/1Ki, V/1Ki) cell lines only supported propagation of the S5-like S segment and two L segments (TSMV-2 and SVaV-2), and the heart- (V/2 Hz), lung- (V/5Lu) and liver-derived (V/1Liv) cell lines allowed propagation of only S5-like S segment, in combination with TSMV-2 L segment (Table 3). S5-like S and TSMV-2 L were also replicated in ball python heart (VI/1 Hz, *P. regius*) and green tree python liver (VII/2Liv, *M. viridis*) cell lines, but not in the carpet python liver cell line (IX/1Liv, *M. spilota*). The S5-like and TSMV-2 pair replicated only in one passage in the green tree python brain cell line (VII/2Br, *M. viridis*), but not in any further passages.

**Table 3.** F15 virus segment detection in SNT after passaging the virus in various cell lines The viruses were passaged a total of five times. The last passage at which each virus segment was detected is shown for each segment in each cell line. Segments that were not detected at all are marked with a dash.

	Family	Boidae						Pythonidae			
	Species	B*. constrictor* (boa constrictor)						*M. viridis* (green tree python)		*M. spilota* (carpet python)	*P. regius* (ball python)
	Tissue origin	Brain	Heart	Lung	Kidney	Kidney	Liver	Brain	Liver	Liver	Heart
	Cell line	V/4Br	V/2 Hz	V/5Lu	I/1Ki	V/1Ki	V/1Liv	VII/2Br	VII/2Liv	IX/1Liv	VI/1 Hz
Virus passaging	S5-like (S)	5	5	5	5	5	5	2	5	–	5
	S6-like (S)	5	5	5	1	–	–	–	–	–	–
	TSMV-2 (L)	5	5	5	5	5	5	–	5	–	5
	SVaV-2 (L)	5	5	5	5	5	1	1	4	1	1
	GMV-1 (L)	–	–	–	1	–	–	–	–	–	1
	ABV-4 (L)	4	5	5	1	–	–	–	–	–	–
	KePV-1 (L)	5	5	5	1	–	–	–	–	–	–
	KMHV-1 (L)	–	–	–	1	–	–	–	–	–	–
	UHV-4 (L)	–	–	–	–	–	–	–	–	–	–

We observed two replicative reptarenavirus segment clusters for the F15 sample. S5-like S and TSMV-2 L segments were always co-enriched together in all cell lines that enabled propagation of the segments, accompanied by SVaV-2 L segment in several boid cell lines (Table 3). Some boid cell lines also showed co-enrichment of ABV-4 and KePV-1 L segments with the S6-like S segment, suggesting some preferred pairing among reptarenavirus segments.

The results of virus passaging with the brain (F17) sample showed all boa constrictor cell lines (V/4Br, V/2 Hz, I/1Ki and V/4Lu) to support replication of all reptarenavirus segments, except for V/1Liv, in which none of the segments were propagated (Table 4). The green tree python and carpet python liver cell lines (VII/2Liv and IX/1Liv) also supported the replication of all reptarenavirus segments; however, the segments propagated only for a single round in the ball python heart cell line (VI/1 Hz). No segments were detected in the green tree python brain (VII/2Br) cell line. The hartmanivirus pair enriched only in the boa constrictor heart (V/2 Hz) cell line.

**Table 4.** F17 virus segment detection in SNT after passaging the viruses and the cells The viruses and cells were passaged a total of five times. The last passage each virus segment was detected is shown for each segment in each cell line. Segments that were not detected at all are marked with a dash.

	Family	*Boidae*					*Pythonidae*			
	Species	*B. constrictor* (boa constrictor)					*M. viridis* (green tree python)		*M. spilota* (carpet python)	*P. regius* (ball python)
	Tissue origin	Brain	Heart	Lung	Kidney	Liver	Brain	Liver	Liver	Heart
	Cell line	V/4Br	V/2 Hz	V/4Lu	I/1Ki	V/1Liv	VII/2Br	VII/2Liv	IX/1Liv	VI/1 Hz
Virus passaging	S7-like (S)	5	5	5	5	–	–	5	5	1
	HJV-1 (L)	5	5	5	5	–	–	5	5	1
	PJV-1 (L)	5	5	5	5	–	–	5	5	1
	KMHV-1 (L)	5	5	5	5	–	–	5	5	1
	MNTV-1 (L)	5	5	5	5	–	–	5	5	1
	VPZV-1 (S)	–	5	–	–	–	–	–	–	–
	VPZV-1 (L)	–	5	–	–	–	–	–	–	–
Cell passaging	S7-like (S)	5	5	5	5	5	–	5	5	–
	HJV-1 (L)	5	5	5	5	–	1	5	1	–
	PJV-1 (L)	5	5	5	5	–	1	5	5	–
	KMHV-1 (L)	5	5	5	5	5	1	5	3	–
	MNTV-1 (L)	5	2	5	5	–	1	–	5	–
	VPZV-1 (S)	–	5	–	–	–	1	5	1	5
	VPZV-1 (L)	–	5	–	–	–	–	5	1	5

Passaging the cells yielded similar results for boid brain, lung, heart and kidney cells, with the minor exception of MNTV-1 that did not replicate in the heart cells (Table 4). Reptarenavirus segment replication and establishment of persistence varied more in python liver cells: the segments were replicated except MNTV-1 in VII/2Liv and HJV-1 and KMHV-1 in IX/Liv cells. No reptarenaviruses were detected in ball python heart (VI/1 Hz) cells during passaging. In addition to the boa constrictor heart (V/2 Hz) cell line, the hartmanivirus also replicated in both green tree python and carpet python liver cell lines (VII/2Liv and IX/1Liv) (Table 4).

## Discussion

Reptarenavirus coinfections in captive snakes represent a rather unique example of the virus–host relationship. Among arenaviruses, the natural/reservoir hosts have only been identified for mammarenaviruses, and the strict associations between each virus species and its respective hosts are assumed to indicate coevolutionary relationships [34]. Others and we have found reptarenaviruses in captive snakes [5,9, 11, 13, 14, 16, 35, 36], and even though we recently found reptarenaviruses in wild boa constrictors native to Central and South America [20,21], there is no definitive proof that snakes are their natural reservoir hosts. Studies on snakes with BIBD have shown that the animals often carry swarms of genetically divergent reptarenavirus L and S segments at unbalanced ratios, the L segments frequently outnumbering the S segment species [8,37]. Curiously, the studies have thus far identified ~30 reptarenavirus L segment species but only ~10 S segment species, suggesting that ~20 S segment species would yet need to be found. The genetic divergence between the segments would support the hypothesis that reptarenaviruses have evolved (each L with a specific S segment) from a common ancestor over tens of thousands of years ago, perhaps together with the currently unknown reservoir host.

**Fig. 2.**
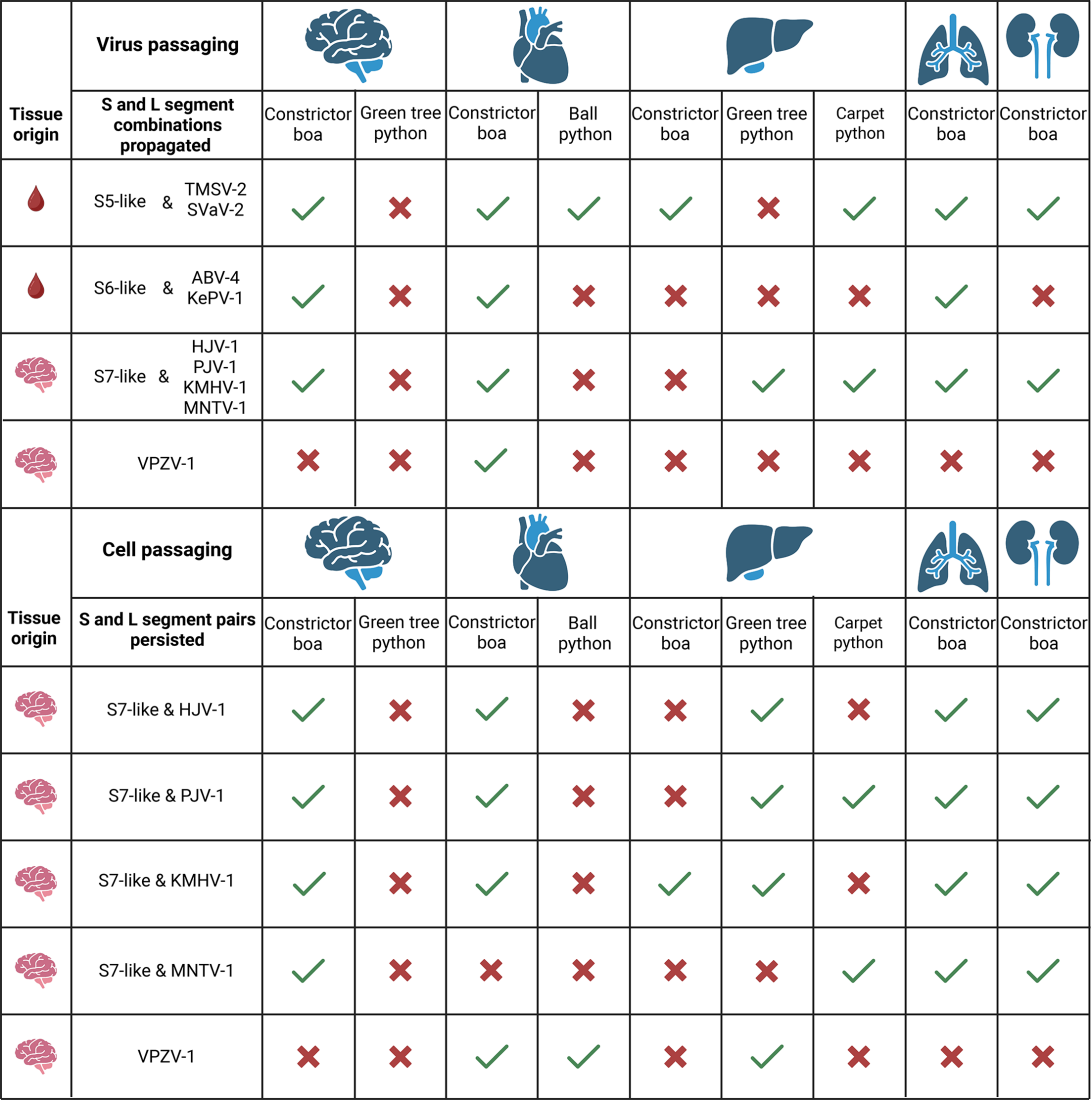
The combinations of co-propagating reptarenavirus S and L segments in the cell lines studied. The segment pairs replicated or persisted in each tissue type of different species are marked with a green checkmark, and with a red cross, they did not replicate. Figure created with BioRender.

Here, we utilized cell lines of some constrictor snakes, families *Boidae* and *Pythonidae*, naturally occurring in Central and South America (boa constrictor, *B. constrictor*), Australasia (green tree python, *M. viridis*, and carpet python*, M. spilota*) and Africa (ball python, *P. regius*), to study whether reptarenavirus S and L segments or their combinations would demonstrate host species preference and/or pairing specificity that could imply coevolutionary relationships. In terms of species tropism, the results showed the boa constrictor-derived brain, heart, lung and kidney cell lines to support replication of several reptarenavirus segments from both blood (F15) and brain (F17). Cells derived from a boa constrictor liver (V/1Liv) supported propagation of only one pair of the reptarenavirus segments present in F15 homogenate, and none of those present in the F17 homogenate when passaging the viruses, implying potential tissue and species tropism associated with the reptarenavirus segments. However, when passaging the V/1Liv cells initially inoculated with the F17 homogenate, we could detect S7-like and KMHV-1 segments after five cell passages. In light of our previous findings that reptarenaviruses are capable of establishing persistent infection in passaged cells [18,19], we propose that establishment of persistent infection in the cell lines would explain or contribute to the observed continued replication (Fig. 2). The tissue origin of the original homogenates F15 and F17 (blood vs. the brain) might also contribute to the observed propagation of the reptarenavirus segments. The fact that some cell lines originating from the family *Pythonidae* supported reptarenavirus replication is in alignment with reports of BIBD in pythons [7,8, 26, 29, 32, 38, 39]. Both green tree python and carpet python liver cell lines (VII/2Liv, *M. viridis*; IX/1Liv, *M. spilota*) supported the replication of all reptarenavirus segments present in the F17 homogenate. However, the green tree python VII/2Liv and ball python heart cell line (VI/1 Hz) supported the propagation of a pair of reptarenavirus segments present in the F15 homogenate, while none of the segments replicated in the carpet python IX/1Liv cell line, supporting the idea of species or tissue tropism (Fig. 2).Interestingly, when employing the virus passaging approach, the hartmanivirus present in the F17 homogenate could only be found in the heart-derived boa constrictor cell line (V/2 Hz). However, when passaging the inoculated cells, the hartmanivirus also amplified in the ball python heart-derived (VI/1 Hz) and green tree python liver-derived (VII/2Liv) cell lines, suggesting that the virus may have established a persistent infection during cell passaging. Passaging of the inoculated cells could allow viruses replicating at lower levels or segment combinations that are less efficient in particle formation to persist in the culture. During passaging, the viruses might employ cell-to-cell spread, e.g. through cell–cell fusion or through utilizing nanotubules. In alignment with the idea of cell-to-cell spread, we found HISV-1 (Haartman Institute snake virus 1, a hartmanivirus) to affect the plasma membrane integrity and to form fluorescent foci in a cell monolayer [16], suggesting that the virus might also employ such a transmission strategy. The observation that all *B. constrictor* cell lines supported at least some reptarenavirus replication, unlike python cells, could relate to the fact that the F15 and F17 homogenates used for inoculations in the study were from BIBD-positive boa constrictors, potentially affecting the segment composition prior to our *in vitro* work. We further interpret the results to indicate that both the tissue and species origin of the cell cultures impact reptarenavirus replication cycle completion. However, the observation that python-derived cell lines allowed propagation of at least some reptarenavirus segment combinations implies that reptarenaviruses would not show host specificity similar to that reported for mammarenaviruses [22,40,42]. The limited number of cell lines per putative host species complicates the interpretation of the results. Unfortunately, growth factors are not available for guiding snake cell line establishment, which we rely on spontaneous immortalization that may have affected the cell type selection.

Our results also provided insights into the pairing of reptarenavirus S and L segments. An earlier study by Stenglein and coworkers provided some evidence of a rather free association between S and L segments [15]. The research on mammarenaviruses indicates that, in addition to the L segment encoded RdRp, their replication requires the expression of NP encoded in the S segment [43,44], thereby providing a mechanistic connection between the segments. In addition to replication, the crucial structural protein interactions between ZP and RdRp, NP and/or GPs play a role in virion formation [45]. On the other hand, the S segment encoded viral GPs mediate the entry of virions into the host cell [46,47], thereby making S segments with suitable GPs essential in mediating tissue and/or species tropism. The results of our experiments showed in the case of the F15 homogenate that there indeed seems to be some tissue and species preference for the enrichment of reptarenavirus S segments, because the S6-like segment only amplified in the heart- and lung-derived boa constrictor cell lines (Fig. 2). Interestingly, the results also revealed preferred amplification of certain L and S segment pairs: S6-like amplified with ABV-4 and KePV-1 L segments and S5-like with SVaV-2 and TSMV-2 L segments (Fig. 2). In two cell lines, only the S5-like S segment and TSMV-2 L segment amplified together, suggesting that this pair would form the most viable combination under these conditions. We suspect that the observed absence of GMV-1, KMHV-1 and UHV-4 could be due to their low segment number in the original sample (Table 1). We speculate the enrichment of certain S and L segment combinations to indicate preferred pairing between segments, arguing against unrestricted reassortment of reptarenavirus segments. Indeed, during the passaging, we found that some segments were lost before passage 5 (Tables3  4). This likely reflects differences in the replication rate between the segments, and it would indeed seem likely that the number of S and L segments gradually declined to a single pair if the passaging were continued long enough. We found our original UHV-1 (University of Helsinki virus 1) stock [5] to actually contain UHV-1 and ABV-1 (aurora borealis virus 1) [14], i.e. two reptarenavirus virus S and L segment pairs. Since then, we have isolated the two viruses, which further supports the idea of preferred S and L segment pairs. The fact that snakes with BIBD most often carry multiple reptarenavirus segments [5,7, 12, 14, 15, 17, 20, 21, 28, 48] could imply that there are cell type and/or tissue-specific differences in the replication rate of different S and L segment combinations.

We recently demonstrated that reptarenaviruses are capable of replicating at temperatures up to 34 °C [49]. This, together with our earlier observation that cell lines originating from various species, including arthropods and vertebrates, allow the replication of reptarenaviruses [37], makes studies on the effect of S and L segment combinations to temperature and species preference an interesting focus of future research. Determining whether certain segments or segment combinations modulate the temperature range of replication could reveal potential host range and BIBD pathogenesis. Such systematic studies could also focus on the contribution of the number and type of S and L segments to BIBD pathogenesis, since S segment RNA levels appear to correlate with the presence of IBs and the number of L segments [48]. To address these questions, future studies should incorporate quantitative approaches such as segment-specific quantitative reverse transcription PCR to allow direct monitoring of viral RNA levels over time, reveal differences in replication dynamics across cell types and clarify the functional compatibility of segment pairings. Here, we took the approach of passaging the virus released from inoculated cells, and we think that the approach results in similar end conclusions, i.e. the viruses with lower RNA amount in the supernatant will gradually be lost during passaging. Such future studies would benefit from the use of reverse genetics that would make S and L segment combinations available; similar systems have been established for mammarenaviruses [50,52] but have not yet been reported for reptarenaviruses. While reptarenaviruses and mammarenaviruses belong to the same family, differences in the replication and molecular biology of the two virus genera cannot be excluded, which has prevented the successful establishment of reverse genetics for reptarenaviruses for now. Nonetheless, the development of such a system remains a promising avenue for future research, as it would enable controlled studies of reassortment and functional segment interactions.

In summary, we interpret the results to suggest that there are preferred reptarenavirus S and L segment combinations, which appear to mediate tissue and species tropism to a certain degree. Our results do not indicate a strict association with a specific host species but rather imply that reptarenaviruses can replicate in cultured cells of various snake species, implying that reptarenaviruses, unlike mammarenaviruses, might not have co-evolved alongside snakes, while their host species remains unknown.

## Supplementary material

10.1099/jgv.0.002154 Uncited Fig. S1.

## Abbreviations

ABV-4: aurora borealis virus 4
BIBD: boid inclusion body disease
GMV-1: grüetzi mitenant virus 1
HJV-1: hipoen jatkoon virus 1
IBs: inclusion bodies
KePV-1: keijut pohjoismaissa virus 1
KMHV-1: kuka mitä häh virus 1
L: large
LCMV: lymphocytic choriomeningitis virus
MNTV-1: mistä näitä tulee virus 1
NP: nucleoprotein
PJV-1: peto jauhoksi virus 1
RdRp: RNA-directed RNA polymerase
S: small
S7-like: S7-like reptarenavirus S segment
SNT: supernatant
SVaV-2: suri vanera virus 2
TSMV-2: tavallinen suomalainen mies virus 2
UHV-4: University of Helsinki virus 4
VPZV-1: Veterinary Pathology Zurich virus 1
ZP: Z protein
