# Supplementary material for: Isolation and passaging of reptarenaviruses utilizing cultured snake cells suggest tissue tropism and restrictions in segment reassortment
Source: J Gen Virol. 2025 Oct 8;106(10):002154. doi: 10.1099/jgv.0.002154 (PMC12507201; doi:10.1099/jgv.0.002154)
Supplement: Uncited Fig. S1. [file jgv-106-02154-s001.pdf]

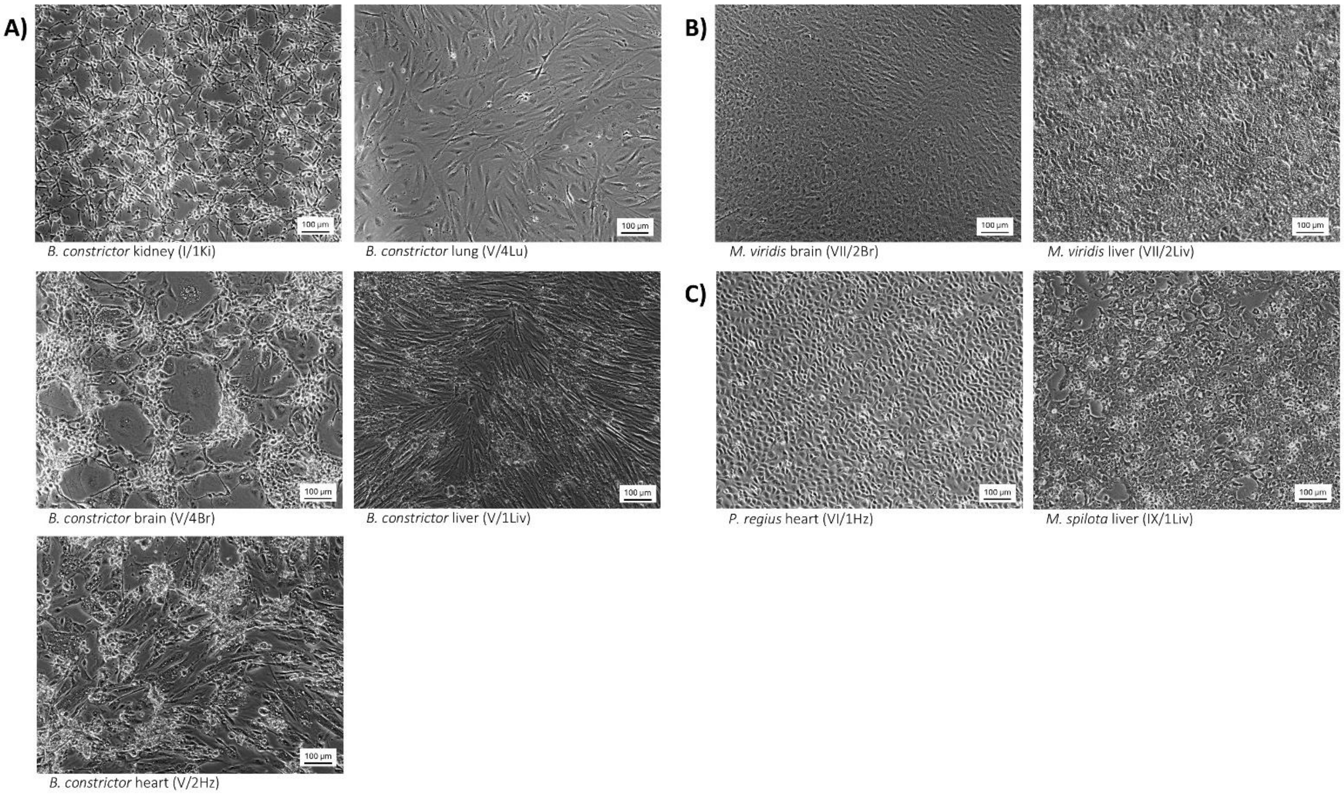

Supplementary Figure 1. Example morphologies of cell lines from each species. Examples of obtained cells from A) B. constrictor and B) M. viridis from previous studies. One cell line per tissue type is shown for kidney and lung cells (I/1Ki vs. V/1Ki; V/4Lu vs. V/5Lu). C) Morphologies of established python cell lines from P. regius and M. spilota. The images were captured with a Nikon Eclipse Ti microscope. Bars = 100 µm.
